# Primary lung cancer with duodenal metastasis complicated by obstructive jaundice and pancreatitis: a case report

**DOI:** 10.3389/fonc.2025.1632696

**Published:** 2025-09-12

**Authors:** Jie Kuang, Yanan Chen, Huikang Xie, Hua Hao, Yanlin Zhao, Cui Tang

**Affiliations:** ^1^ Department of Radiology, Yangpu Hospital, School of Medicine, Tongji University, Shanghai, China; ^2^ Department of Radiology, Shanghai Pulmonary Hospital, Tongji University School of Medicine, Shanghai, China; ^3^ Department of Pathology, Shanghai Pulmonary Hospital, Tongji University School of Medicine, Shanghai, China; ^4^ Department of Pathology, Yangpu Hospital, School of Medicine, Tongji University, Shanghai, China

**Keywords:** lung cancer duodenal metastasis, rare metastasis, obstructive jaundice, pancreatitis, case report

## Abstract

**Background:**

Primary lung adenocarcinoma with duodenal metastasis is relatively rare, and its early clinical symptoms are often insidious, making diagnosis challenging. CT, MR, PET-CT, and gastroscopy can effectively identify gastrointestinal metastatic lesions, providing reliable evidence for definitive diagnosis and treatment planning, which contributes to prolonging patient survival.

**Case presentation:**

This report presents a rare case of duodenal metastasis from lung adenocarcinoma. The patient was diagnosed with lung adenocarcinoma (cT4N3M1c, stage IVb) one year prior. During chemotherapy, the patient gradually developed symptoms of abdominal distension and progressive jaundice. Through analysis of CT and MR imaging changes during the disease course, combined with laboratory test indicators, malignant duodenal tumor causing biliary obstruction and pancreatitis was suspected. Ultimately, endoscopic pathological biopsy confirmed duodenal metastasis from primary lung cancer. The clinical surgeon implemented a PTCD treatment plan, successfully relieving the patient’s biliary obstruction.

**Conclusions:**

Primary lung cancer patients presenting with abdominal symptoms and imaging findings suggestive of gastrointestinal tumors should be highly suspected of metastasis. Timely pathological diagnosis is essential to determine the nature and origin of the tumor, thereby facilitating the formulation of individualized treatment plans.

## Introduction

According to the GLOBOCAN 2022 report by the International Agency for Research on Cancer (IARC) of the World Health Organization, primary lung cancer is the malignancy with the highest incidence and mortality rates globally (1). The predominant metastatic sites of this malignancy encompass the brain, liver, adrenal glands, and skeletal system (2–8). In clinical practice, gastrointestinal metastasis of lung carcinoma is infrequently observed, largely attributable to the fact that the majority of patients do not undergo definitive diagnostic confirmation throughout the disease progression. Studies indicate that the probability of gastrointestinal metastasis in lung cancer ranges from 0.3% to 1.77% (4, 5, 9), which is consistent with autopsy statistics. In actual clinical practice, the number of such cases may be significantly higher than currently reported. Therefore, early diagnosis through imaging examinations, endoscopy, and histopathological analysis is essential to guide clinical treatment. This article reports a case of primary lung adenocarcinoma with duodenal metastasis, accompanied by biliary obstruction and pancreatitis. The imaging findings are analyzed to provide a reference for the formulation of clinical treatment strategies.

## Case presentation

**Figure 1:**
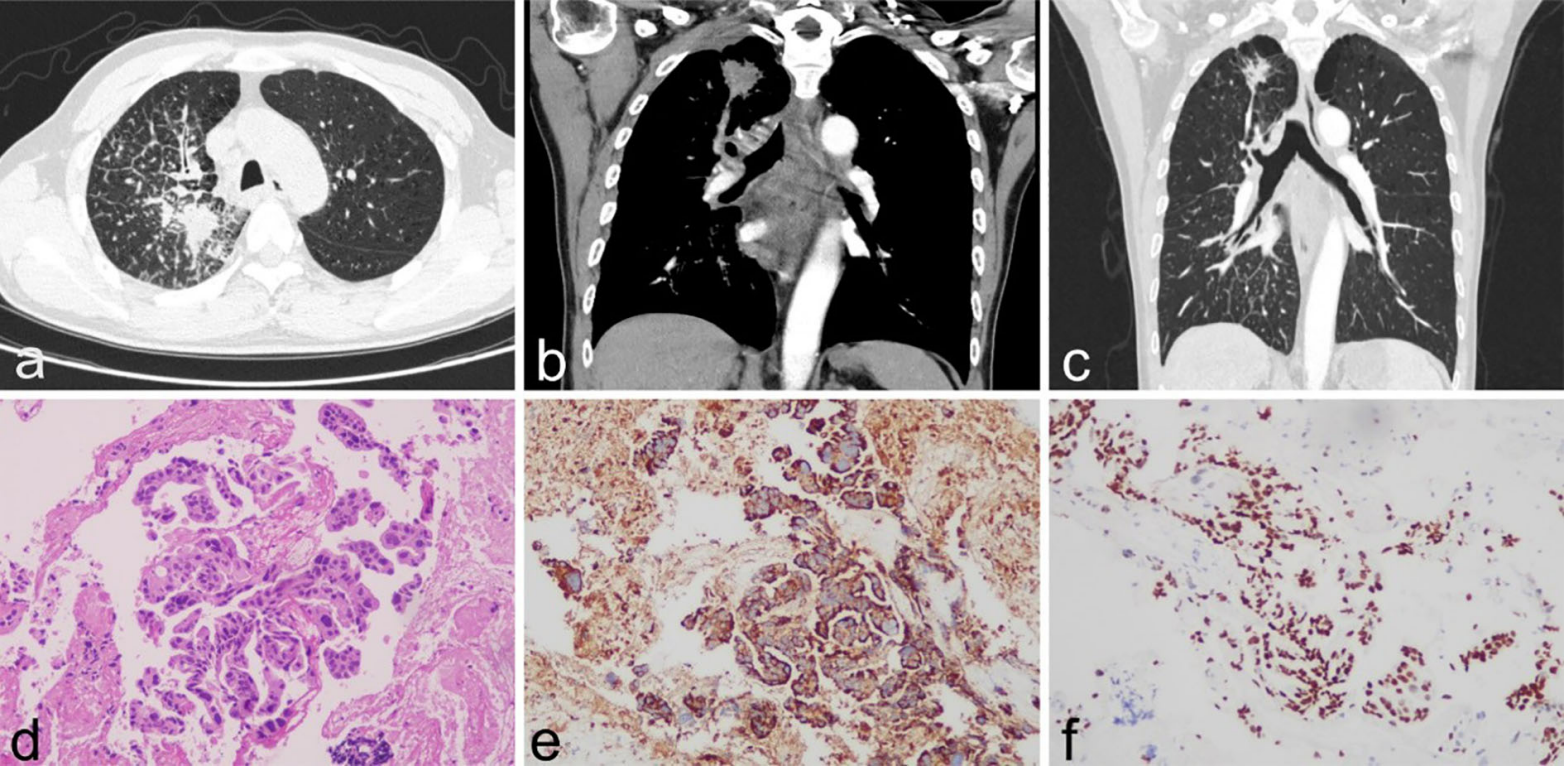
**(a–c)** Contrast-enhanced CT findings. **(a, b)** (Institutional CT, February 20, 2024): A right upper lobe lung mass with obstructive pneumonitis and lymphangitis carcinomatosa. **(c)** (External institution CT, October 9, 2024): Significant tumor regression in the right upper lobe. **(d)** presents the HE-stained section of the lung biopsy, which reveals glandular tumor cells, indicative of adenocarcinoma. **(e, f)** IHC showing NapsinA (+), TTF-1(+).

A 61-year-old male patient was diagnosed with stage IVb right lung adenocarcinoma (cT4N3M1c, involving brain and cervical lymph nodes) harboring a KRAS G12C mutation one year prior (**Figures 1d–f**). On February 20, 2024, contrast-enhanced chest CT imaging at our institution demonstrated a right upper lobe lung mass with associated localized obstructive inflammation and carcinomatous lymphangitis, accompanied by multiple enlarged lymph nodes in the mediastinum and right hilum (**Figures 1a, b**). The patient had previously received systemic therapy at an external facility, comprising a combination of immunotherapy (AK104, a PD-1/CTLA-4 bispecific antibody [cadonilimab], and AK112, a PD-1/VEGF bispecific antibody) and palliative chemotherapy (carboplatin plus pemetrexed), totaling 11 treatment cycles, during which carboplatin administration was discontinued. Follow-up contrast-enhanced CT imaging at the external hospital revealed a reduction in the size of the right upper lobe tumor and mediastinal lymph nodes (**Figure 1c**).

**Figure 2:**
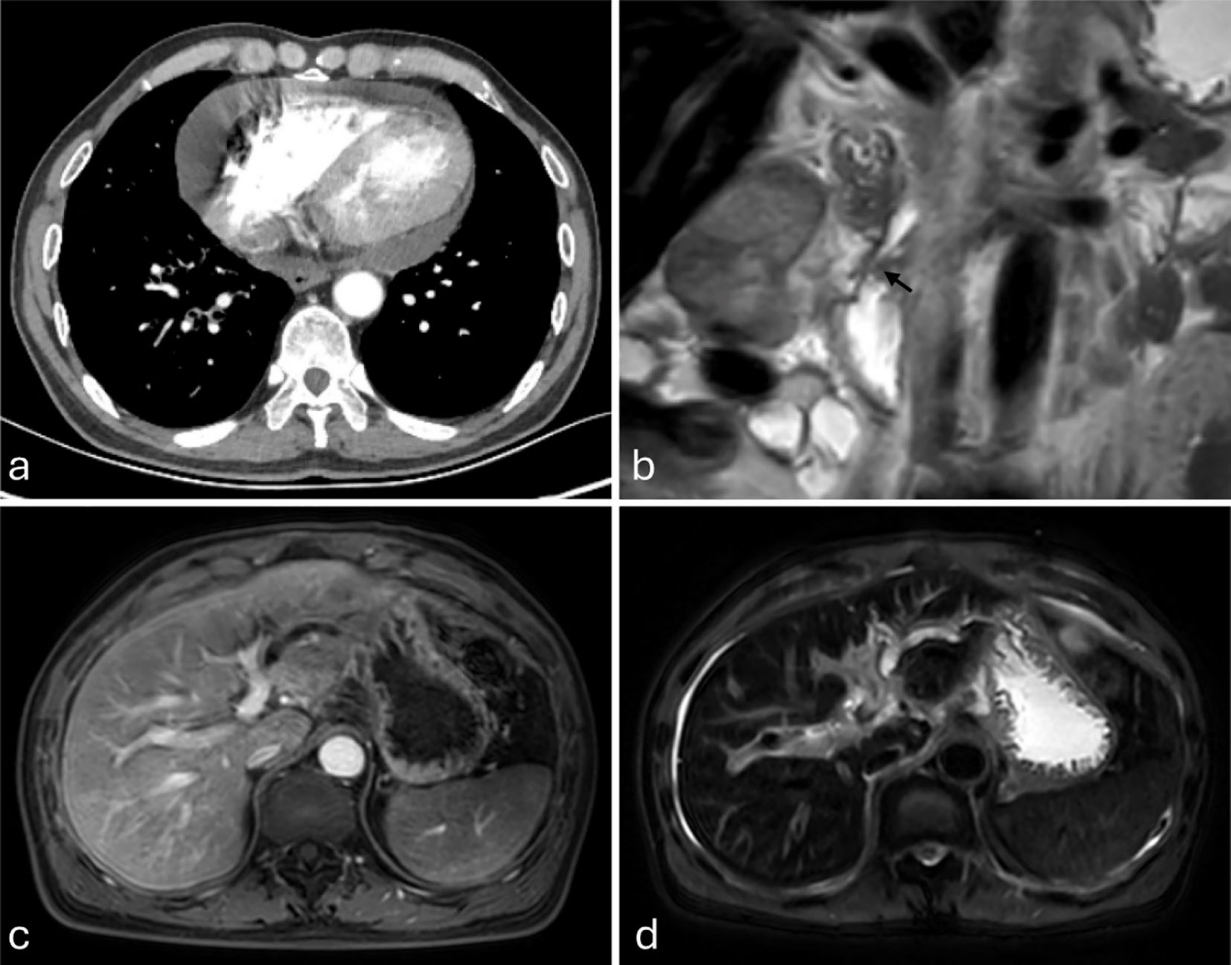
**(a–d)** Imaging studies from an external institution (December 5, 2024). **(a)** Contrast-enhanced chest CT demonstrates pericardial effusion. **(b–d)** Magnetic resonance imaging (MRI), **(b)** Coronal T2-weighted imaging (T2WI) clearly demonstrates tapered stenosis at the distal common bile duct and pancreatic duct orifice (marked by black arrows). **(c)** Axial fat-suppressed contrast-enhanced T1-weighted imaging (T1WI) shows intrahepatic biliary dilatation in the left hepatic lobe. **(d)** Axial fat-suppressed T2WI demonstrates left hepatic lobe intrahepatic biliary dilatation and ascites.

On December 5, 2024, pericardial effusion was identified in the patient during the course of treatment at an external medical facility (**Figure 2a**). A pericardiocentesis procedure was performed, yielding approximately 1000 ml of hemorrhagic effusion. Subsequent cytological analysis of the specimen confirmed the presence of malignant cells, consistent with adenocarcinoma, alongside elevated serum levels of γ-glutamyl transpeptidase and alkaline phosphatase (**Table 1**). Magnetic resonance imaging (MRI) of the liver conducted at the external hospital revealed intrahepatic bile duct dilation and ascites (**Figures 2b-d**). In response to these findings, the therapeutic strategy was modified to a combination of chemotherapy and immunotherapy, comprising nab-paclitaxel (400 mg administered on day 1) and camrelizumab (200 mg administered on day 1).

**Table 1:** Summary table of biochemical indicators for patients.

Date	TBIL (μmol/L)	DBIL (μmol/L)	IBIL (μmol/L)	γ-GT (U/L)	ALP (U/L)	ALT (U/L)	AST (U/L)	LPS (U/L)	AMY (U/L)
2024/12/5	–	–	–	264.7	198	–	–	–	–
2025/1/6	–	–	–	525.2	347.2	–	–	–	–
2025/2/6	133.3	122	11.3	1357.7	908.6	232.2	103.7	–	–
2025/2/8	162.9	132.2	30.7	1045	741	197	83	1143	157
2025/2/10	199.2	123.4	75.8	889	704	161	80	134	93
2025/2/12	212.6	158.3	54.3	748	571	123	75	88	64
2025/2/15	269.2	213.7	55.5	1171	666	185	142	90	57

“-” indicates a normal indicator.

**Figure 3:**
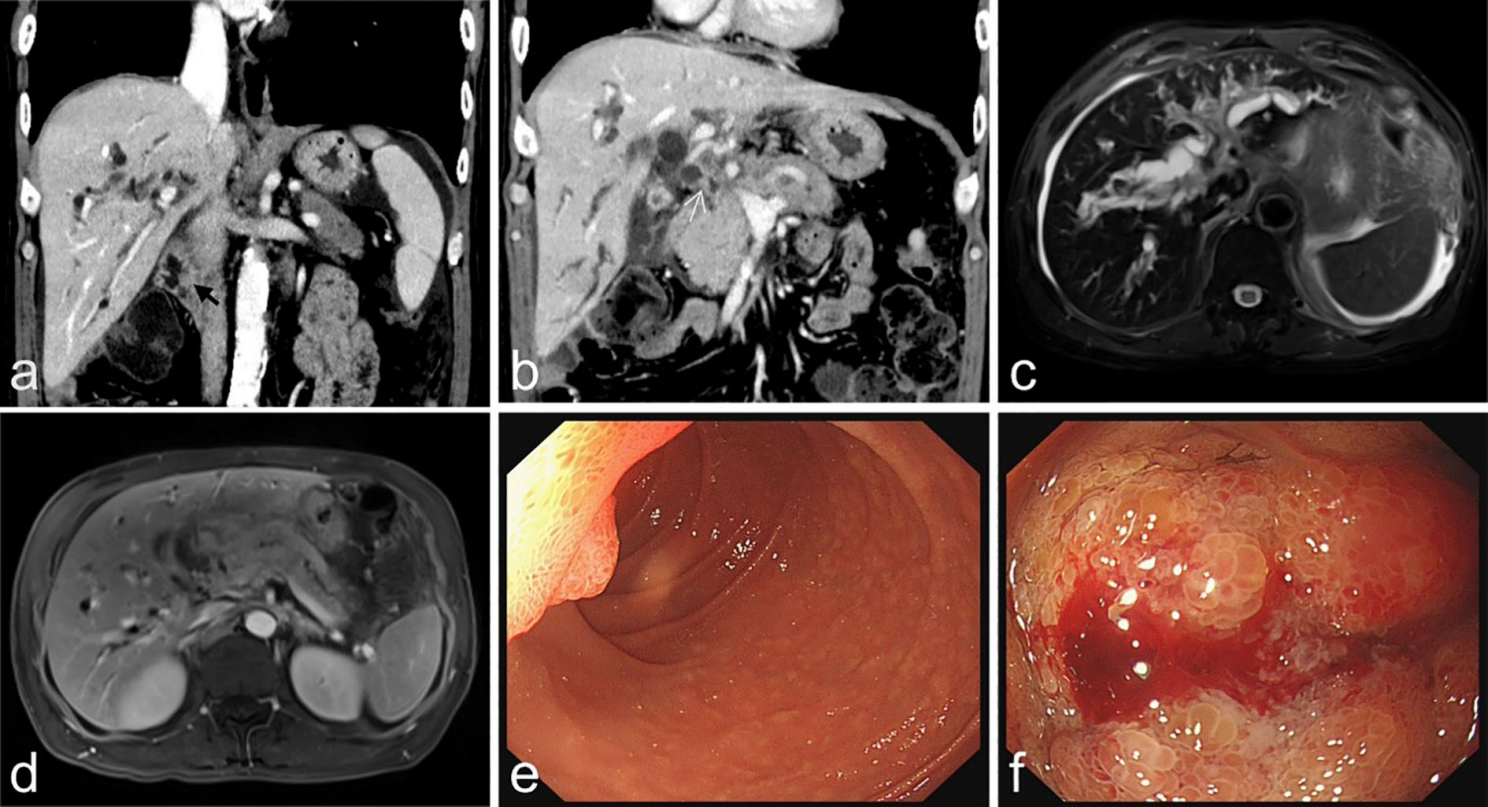
**(a, b)** (Institutional contrast-enhanced CT, venous phase, coronal reconstruction; February 10, 2025) demonstrate the following findings: **(a)** A small, abnormally enhancing nodule (indicated by a black arrow) localized at the duodenal papilla, accompanied by significant dilatation of the intrahepatic bile ducts and the proximal common bile duct. **(b)** Focal mural thickening with heterogeneous enhancement in the distal common bile duct (marked by a white arrow), alongside diffuse enlargement of the pancreatic body and tail. Figures **(c, d)** (Institutional pancreatic MRI; February 11, 2025) reveal: **(c)** Axial fat-suppressed T2-weighted imaging (T2WI) illustrating the “soft rattan sign,” indicative of intrahepatic biliary dilatation. **(d)** Axial fat-suppressed T1-weighted contrast-enhanced (venous phase) imaging showing pancreatic duct dilatation and pancreatic parenchymal enlargement. Figures **(e, f)** present esophagogastroduodenoscopy (EGD) findings: **(e)** A proliferative lesion with an irregular/nodular surface is identified at the major duodenal papilla in the descending duodenum. **(f)** Marked mucosal edema and surface coarsening/granularity are observed in the duodenal bulb.

**Figure 4:**
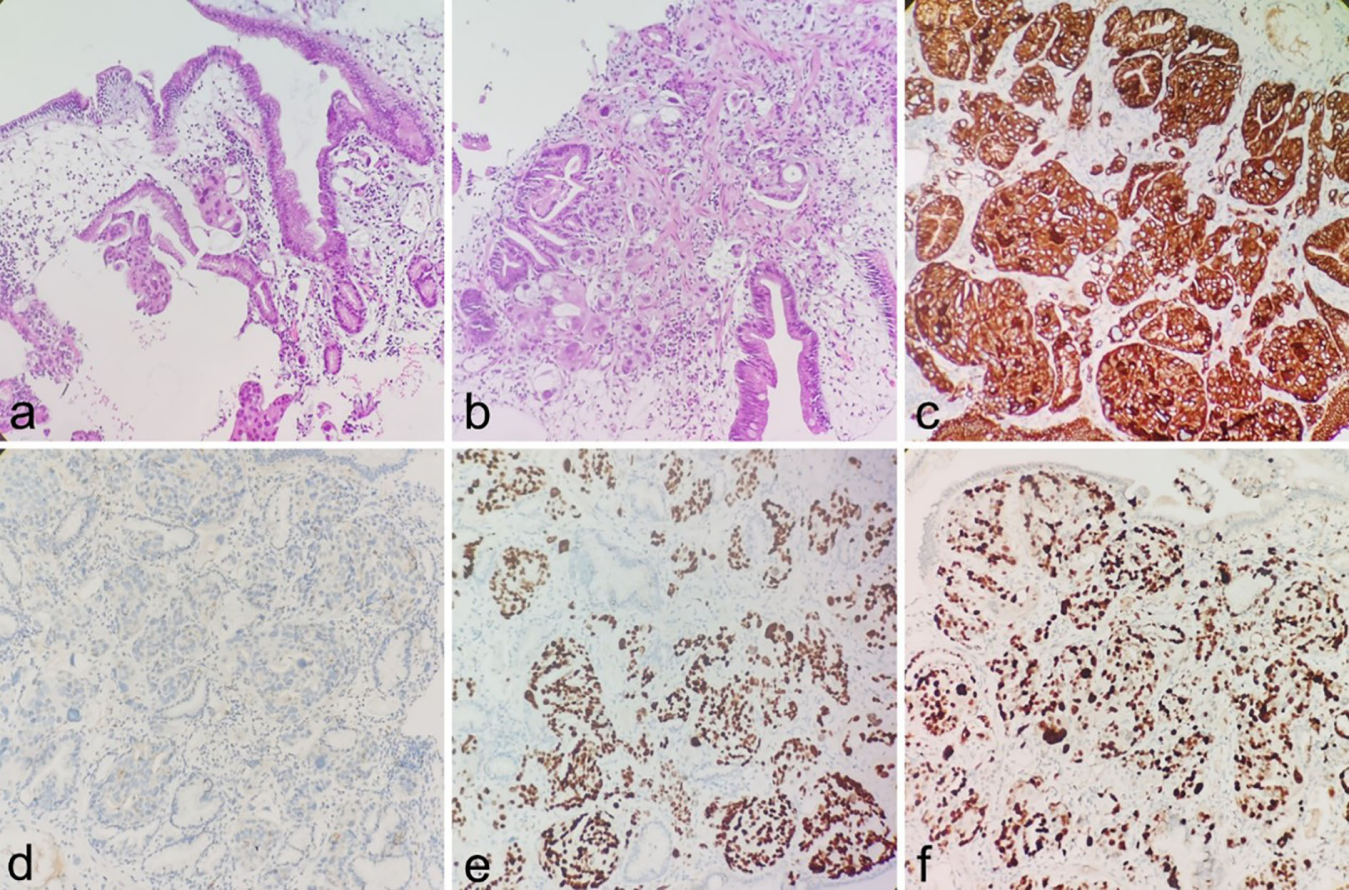
**(a, b)** Infiltration of adenocarcinoma is observed within the duodenal bulb and periampullary stroma (H&E). **(c–f)** IHC showing CK-pan(+), NapsinA (focal+), TTF-1(+), The Ki-67 proliferation index of tumor cells was approximately 60%.

Following discharge, the patient underwent regular liver function monitoring. Two months post-discharge, clinical manifestations including anorexia, abdominal distension, dark-colored urine, and lower back pain emerged, accompanied by progressive jaundice exacerbation. Biochemical analyses revealed persistent deterioration of hepatic function (**Table 1**), with elevated tumor markers: CEA (93.01 ng/ml), CA125 (59.51 U/ml), CA153 (78.37 U/ml), and CYFRA21-1 (16.75 ng/ml). On February 10, 2025, contrast-enhanced abdominal CT demonstrated marked dilation of intrahepatic bile ducts and the proximal common bile duct, with stenosis observed in the mid-to-distal common bile duct and heterogeneous post-contrast enhancement. Pancreatic body and tail thickening were noted. Subsequent pancreatic MRI revealed pancreatic fullness with outward bulging of the head and neck contours, accompanied by abnormal signal intensity. Intrahepatic and common bile ducts exhibited vine-like dilation, with concurrent pancreatic duct dilation (**Figures 3a–d**). Radiological assessment suggested biliary and pancreatic duct obstruction, with suspicion of a pancreatic head/neck mass. Following multidisciplinary consultation, the therapeutic strategy included (1): serial monitoring of liver function and pancreatitis indicators (2); PTCD implementation in cases of biliary obstruction with infection or disease progression to facilitate biliary drainage and prevent hepatic failure; and (3) endoscopic evaluation of the duodenal papilla (**Figures 3e, f**). Definitive diagnosis of metastatic adenocarcinoma in the duodenal papilla and bulb was established through endoscopic biopsy with immunohistochemical analysis (**Figure 4**).

On February 19, 2025, percutaneous transhepatic cholangiodrainage (PTCD) was performed under intravenous anesthesia to facilitate bile drainage, accompanied by comprehensive symptomatic management including antimicrobial therapy, jaundice reduction protocols, and nutritional supplementation. Following six days of treatment, the patient demonstrated significant clinical improvement, leading to an uneventful discharge.

## Discussion

The gastrointestinal tract is an uncommon site for metastasis of primary lung cancer (5, 10), with adenocarcinoma having the lowest probability of gastrointestinal metastasis among different histological types of lung cancer (11). Clinical manifestations typically include abdominal masses, abdominal pain, intestinal obstruction or intussusception caused by the mass, gastrointestinal perforation, and bleeding (3, 4, 6, 8, 11–17). In this case, the patient presented with duodenal metastasis from primary lung adenocarcinoma, with clinical symptoms of decreased appetite, abdominal distension, and progressive jaundice, which is rare compared to previous literature reports (18).

Eight months post-chemotherapy, the patient underwent contrast-enhanced abdominal computed tomography (CT), which revealed no significant abnormalities. However, subsequent contrast-enhanced CT and magnetic resonance (MR) imaging performed one month later demonstrated intrahepatic bile duct dilation and ascites, prompting a diagnosis of liver injury based on biochemical markers. In the imaging examination conducted one month later, we initially did not identify any definitive mass on the initial review of the images. However, given the progression of biliary obstruction (manifested as the “soft vine sign” in the biliary system), significant dilation of the pancreatic duct, and heterogeneous enhancement of the pancreas, we suspected the presence of a space-occupying lesion at the pancreaticobiliary duodenal junction.

Upon re-examining the imaging, we identified a small enhancing nodule at the duodenal papilla (**Figure 3a**), along with mucosal thickening and abnormal enhancement in the duodenal bulb. Subsequently, we recommended an endoscopic examination to further clarify the nature of the lesions in the duodenal papilla and bulb. Although the patient’s imaging showed a prominent “double duct sign,” (19, 20), the pancreas appeared thickened with heterogeneous enhancement and peripancreatic exudation. Given the patient’s history of immunotherapy, we considered the possibility of treatment-related acute pancreatitis or obstructive pancreatitis.

In the diagnostic evaluation, comprehensive consideration of biochemical parameters and tumor marker profiles is essential (19–22). Notably, the patient’s serum CA-199 levels remained within normal limits, which provided additional supportive evidence for the radiological findings. The definitive diagnosis of metastatic adenocarcinoma involving the duodenal papilla and bulb was established through histopathological examination of gastroscopic biopsy specimens, with confirmation via immunohistochemical analysis.

Research has shown that patients with lung cancer with gastrointestinal metastases have poor prognosis (5, 15, 23), mainly due to the high invasiveness and rapid progression of metastatic lesions, and often delayed clinical diagnosis. At present, the diagnosis of gastrointestinal metastasis relies mainly on gastroenteroscopy and pathological immunohistochemical analysis (10, 16, 24–26). However, some patients are not suitable for gastroenteroscopy because of their physical condition, and abdominal symptoms are easily overlooked in clinical practice, resulting in inadequate endoscopy. Therefore, clinicians often use imaging examinations such as CT and MR as the main means of follow-up and screening, which have the advantages of non-invasive and reproducibility, and can provide important basis for early diagnosis.

CT plays a crucial role in identifying the causes of abdominal pain in lung cancer patients and assessing gastrointestinal metastasis. Studies have shown that the sensitivity of CT scans in detecting gastrointestinal metastasis of lung cancer can reach 93% (3). Typical imaging features include: focal or segmental thickening of the intestinal wall, exophytic or intraluminal polypoid masses (4, 7, 12), irregular mucosa or ulcers (3), local lymph node enlargement, and intestinal perforation or intussusception (27, 28). Enhancement patterns vary, with moderate enhancement being the most common (28). Notably, performing CT examination with the duodenum fully distended and filled with fluid can significantly improve the detection rate of duodenal lesions (29).

Magnetic resonance (MR) imaging is less commonly employed in the detection of gastrointestinal metastases from lung cancer, primarily due to its extended acquisition time and the heightened requirement for patient compliance. However, MR imaging serves as a valuable diagnostic tool for elucidating the underlying etiology, enabling detailed visualization of biliary obstruction, and excluding the presence of additional space-occupying lesions at the pancreaticoduodenal junction.

Positron Emission Tomography-Computed Tomography (PET-CT) has been established as a highly effective diagnostic modality for identifying subclinical gastrointestinal (GI) metastases in lung cancer patients. Furthermore, it plays a critical role in the comprehensive evaluation of extrapulmonary metastases and serves as an essential tool for the initial staging of small intestinal metastases in cases of non-small cell lung cancer (NSCLC) (2, 4, 16, 30). Nevertheless, this imaging technique is not advised for patients presenting with acute symptoms.

To date, standardized therapeutic protocols for gastrointestinal metastasis of lung cancer remain undefined, with early detection being a critical determinant of favorable treatment outcomes. In the present case, the patient manifested progressive abdominal pain and jaundice over a one-year treatment course. Through comprehensive analysis of serial CT and MR imaging findings in conjunction with laboratory parameters, radiologists accurately identified biliary obstruction and pancreatitis secondary to a duodenal tumor, thereby providing crucial diagnostic insights for clinical management. The imaging-based diagnosis was subsequently confirmed by endoscopic pathological biopsy, enabling clinicians to implement targeted interventions, including percutaneous transhepatic cholangial drainage (PTCD), which effectively ameliorated the patient’s clinical symptoms.

## Conclusion

In conclusion, clinicians should maintain a high level of vigilance for gastrointestinal metastasis in lung cancer patients, particularly when patients present with unexplained gastrointestinal symptoms. Prompt imaging studies and gastrointestinal endoscopy should be conducted. If a duodenal mass is detected, the possibility of metastasis should be considered. For patients diagnosed with duodenal metastasis, attention should be paid to clinical manifestations such as biliary obstruction and pancreatitis. Early diagnosis facilitates the formulation of personalized treatment plans, thereby improving patient prognosis and quality of life.

## Data Availability

The original contributions presented in the study are included in the article/supplementary material. Further inquiries can be directed to the corresponding author.
